# Low tidal volume pressure support versus controlled ventilation in early experimental sepsis in pigs

**DOI:** 10.1186/s12931-014-0101-6

**Published:** 2014-09-06

**Authors:** Alexander Ziebart, Erik K Hartmann, Rainer Thomas, Tanghua Liu, Bastian Duenges, Arno Schad, Marc Bodenstein, Serge C Thal, Matthias David

**Affiliations:** Department of Anesthesiology, Medical Centre of the Johannes Gutenberg-University, Langenbeckstr. 1, 55131 Mainz, Germany; Institute of Pathology, Medical Centre of the Johannes Gutenberg-University, Langenbeckstr. 1, 55131 Mainz, Germany

**Keywords:** ARDS, Sepsis-induced lung injury, Pressure support ventilation, Volume controlled ventilation, Pig model

## Abstract

**Background:**

In moderate acute respiratory distress syndrome (ARDS) several studies support the usage of assisted spontaneous breathing modes. Only limited data, however, focus on the application in systemic sepsis and developing lung injury. The present study examines the effects of immediate initiation of pressure support ventilation (PSV) in a model of sepsis-induced ARDS.

**Methods:**

18 anesthetized pigs received a two-staged continuous lipopolysaccharide infusion to induce lung injury. The animals were randomly assigned to PSV or volume controlled (VCV) lung protective ventilation (tidal volume each 6 ml kg^-1^, n = 2x9) over six hours. Gas exchange parameters, hemodynamics, systemic inflammation, and ventilation distribution by multiple inert gas elimination and electrical impedance tomography were assessed. The post mortem analysis included histopathological scoring, wet to dry ratio, and alveolar protein content.

**Results:**

Within six hours both groups developed a mild to moderate ARDS with comparable systemic inflammatory response and without signs of improving gas exchange parameters during PSV. The PSV group showed signs of more homogenous ventilation distribution by electrical impedance tomography, but only slightly less hyperinflated lung compartments by multiple inert gas elimination. Post mortem and histopathological assessment yielded no significant intergroup differences.

**Conclusions:**

In a porcine model of sepsis-induced mild ARDS immediate PSV was not superior to VCV. This contrasts with several experimental studies from non-septic mild to moderate ARDS. The present study therefore assumes that not only severity, but also etiology of lung injury considerably influences the response to early initiation of PSV.

## Background

In the course of non-pulmonary sepsis respiratory failure is a common cause and occurs in about 50% of the patients with severe sepsis [1]. Patients suffering from sepsis often require mechanical ventilation, even if they do not fulfill the criteria of an acute respiratory distress syndrome (ARDS). On the other hand, mechanical ventilation itself can represent the second hit leading to the development of ARDS. Independent from the underlying illness, lung protective strategies that aim to minimize ongoing pulmonary damage by targeting low tidal volumes and limitation of the inspiratory pressure are regarded as the key interventions when the criteria of ARDS are met [2,3]. In severe ARDS there is evidence that short-term neuromuscular blockade enables the consequent realization of low tidal volume ventilation and increases survival rates [4]. In mild to moderate or post-acute ARDS, however, the admittance of spontaneous breathing is reported to improve gas exchange, reduce diaphragmatic dysfunction and enable a faster weaning [5,6]. Several experimental models report beneficial effects of spontaneous breathing in various patterns on gas exchange, edema formation, and lung injury [7–9]. These findings, though, have not been verified in primary sepsis-related lung injury. Furthermore, some clinical and experimental data also suggest the value of preventive initiation of lung protective ventilation [10]. But currently the appropriate guidelines do not state on the preemptive application of lung protective ventilation or spontaneous breathing in early sepsis-induced lung injury [11].

We hypothesized that in early sepsis immediate application of pressure support ventilation (PSV) targeted to a tidal volume (V_t_) of 6 ml kg^-1^ will improve the pulmonary function in comparison to conventional volume controlled ventilation (VCV, V_t_ 6 ml kg^-1^). Hence, we compared the early effects of PSV and VCV on gas exchange, ventilation/perfusion distribution and histopathological lung injury in a porcine model of systemic, lipopolysaccharide (LPS)-induced sepsis subsequently leading to lung injury.

## Methods

The study was approved by the State and Institutional Animal Care Committee (Landesuntersuchungsamt Rheinland-Pfalz, Koblenz, Germany; approval number: G10-1-004). 18 juvenile pigs (Sus scrofa domestica, weight 27 ± 2 kg) were examined in a prospective-randomized setting.

### Anesthesia and instrumentation

The animals were sedated by an intramuscular injection of ketamine (8 mg kg^.1^) and midazolam (0.2 mg kg^-1^). Anesthesia was induced by intravenous application of propofol (4 mg kg^-1^) and fentanyl (4 μg kg^-1^). A single shot of atracurium (0.5 mg kg^-1^) was added to facilitate endotracheal intubation (internal diameter 7.5 mm tube). General anesthesia was maintained by infusion of ketamine (10-20 mg kg^-1^ h^-1^) and midazolam (0.5-2 mg kg^-1^ h^-1^). Volume controlled ventilation (VCV; AVEA, CareFusion, USA) was used during the preparation period: V_t_ 6 ml kg^-1^, positive end-expiratory pressure (PEEP) 7 cmH_2_O, fraction of inspired oxygen (FiO_2_) 0.35, variable respiratory rate to guarantee an endtidal CO_2_ (etCO_2_) < 8 kPa, and ph >7.2. Vascular catheters were placed ultrasound-guided in Seldinger’s technique by femoral access: a central venous line, a pulmonary arterial catheter and a PiCCO®-System (Pulsion Medical Systems, Germany). Spirometry and hemodynamics were permanently stored (Datex S/5, GE Healthcare, Germany). The esophageal pressure was measured with an esophageal balloon catheter. Body temperature was measured by a rectal probe, while a surface-warming device maintained normothermia.

### Experimental protocol

**Figure 1:**
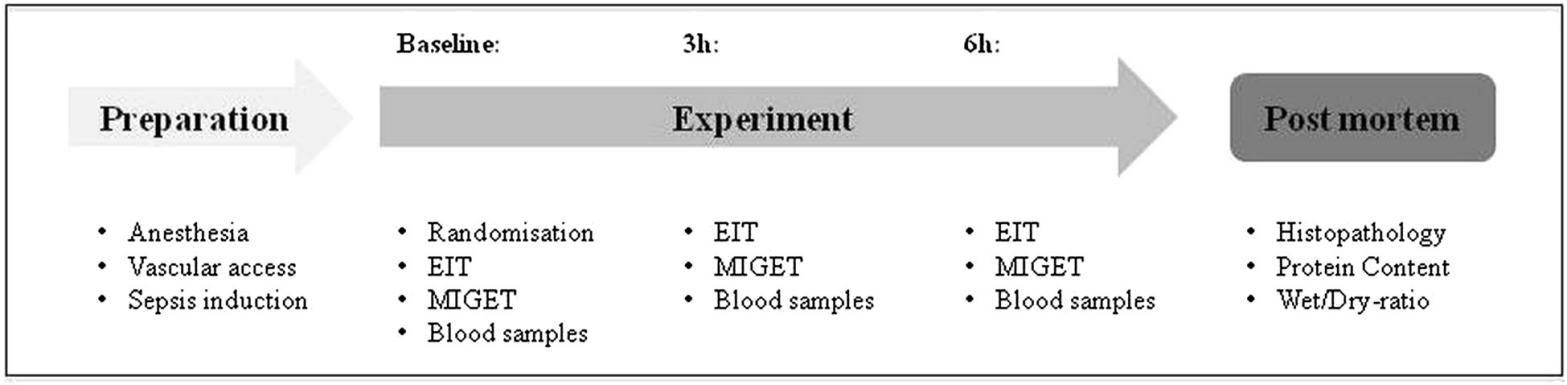
**Experimental protocol.** EIT: electrical impedance tomography, MIGET: multiple inert gas elimination technique.

Figure 1 summarizes the experimental protocol. Septic inflammatory response was induced by continuous LPS infusion (E. coli Serotype O111:B4, Sigma-Aldrich, Switzerland). The infusion scheme includes a high-dose induction (100 μg kg^-1^ h^-1^) over one hour and a maintenance dosage (10 μg kg^-1^ h^-1^) for the entire experiment. Following anesthesia and preparation, but before sepsis induction a non-participant randomized the animals by drawing one of 18 envelopes containing the respective ventilation mode:

- **PSV-Mode (n = 9):** pressure support 15 ± 5 cmH_2_O, V_t_ 6 ml kg^-1^, PEEP 5 cmH_2_O, FiO_2_ 0.35, trigger = 1.5 l min^-1^ targeted to an etCO_2_ < 8 kPa and ph > 7.2
- **VCV-Mode (n = 9):** V_t_ 6 ml kg^-1^, PEEP 5 cmH_2_O, FiO_2_ 0.35, variable respiratory rate targeted to an etCO_2_ < 8 kPa and ph > 7.2

The VCV group received repeated injections of atracurium (0.5 mg kg^-1^) to avoid the onset of spontaneous breathing under close monitoring of depth of anesthesia. Measurements were performed at baseline, three and six hours after sepsis induction. To prevent severe hypoxemia or hypercapnia during LPS infusion and developing lung injury that would lead to implausible results, we established an intervention scheme instead of using a fixed setting, which was oriented on the ARDS Network PEEP/FiO_2_ tables: if the peripheral oxygen saturation dropped under 92% for five minutes, the ventilation parameters were adapted. During the experiment a balanced saline solution (5 ml kg^-1^ h^-1^; Sterofundin, B. Braun Germany) was applied continuously. In case of hemodynamic instability (mean arterial pressure < 60 mmHg) the animals received a hydroxylethyl starch infusion (90 ml h^-1^; Volulyte 6%, Fresenius Kabi, Germany) and an additional bolus once per hour. Persisting instability was treated by continuous noradrenaline infusion. The animals were monitored over six hours following sepsis induction. At the end of the experiments the animals were killed in deep general anesthesia by intravenous injection of propofol (200 mg) and exsanguination.

### Electrical impedance tomography and multiple inert gas elimination technique

A 16-electrode electrical impedance tomography device (EIT; Goe-MF II, CareFusion, Germany) recorded relative bioimpedance changes related to pulmonary aeration. To analyze the regional ventilation distribution, the percentage of the respiratory-dependent relative impedance changes was attributed to three regions of interest (non-dependent, central, dependent). Setup, data acquisition and processing were previously described in detail [12,13]. The ventilation/perfusion (V_A_/Q) distribution was assessed by means of micropore membrane inlet mass spectrometry - multiple inert gas elimination technique (MMIMS-MIGET, Oscillogy LLC, USA) [14,15].

### Post mortem and histopathological analysis

The lungs were extracted en bloc under continuous positive airway pressure. The upper left lobe was used for bronchoalveolar lavage to determine the alveolar protein content. The lower left lobe was weighted and dried to measure the wet to dry ratio. The right lung was used to quantify the histopathological damage oriented on established scoring systems [16]. The assessment was performed in investigator-blinded manner under supervision of an experienced pathologist. Representative samples of different regions (non-dependent, central, dependent) were extracted and fixed in formalin for paraffin sectioning and hematoxylin/eosin staining. The evaluation included seven different parameters: overdistension, epithelial destruction, inflammatory infiltration, alveolar edema, hemorrhage, interstitial edema, and microatelectasis. Per region each parameter received a severity grade from zero to five points in four non-overlapping fields of view. In a second step the extent of each parameter was assessed in a global overview of the entire region. This results in a summarized score of 175 maximum points per region. Plasma levels of inflammatory cytokines (IL-6, TNF-α) were determined by means of enzyme-linked immunosorbent assays (Porcine IL-6 Quantikine ELISA; Porcine TNF-alpha Quantikine ELISA, R&D System, Germany). Lactate, thrombocytes and leukocytes were analyzed by the Institute of Laboratory Medicine, University Medical Centre Mainz.

### Statistical analysis

Data are reported as mean and standard deviation (SD) or box-plots. Baseline values were compared by t-test or Mann-Whitney-U-Test depending on presence of Gaussian distribution. The effects of group (PSV vs. VCV) and group over time were assessed by two-way analysis of variance (ANOVA) and post-hoc Holm-Sidak-Test. Post mortem data were examined by Mann-Whitney-U-Test and adjusted for multiple comparisons by the Bonferroni method. P values < 0.05 are regarded as significantly different. The statistical software SigmaPlot 12.5 (Systat Software, Germany) was used.

## Results

The study protocol was completed in all 18 animals. Table 1 summarizes the ventilatory and hemodynamic data and shows no intergroup differences at baseline.

**Table 1:** **Respiratory and haemodynamic data**

**Variables**	**VCV**			**PSV**			**Group effect**
	**Baseline**	**3 h**	**6 h**	**Baseline**	**3 h**	**6 h**	
FiO_2_ [%]	36 ± 1	47 ± 13	56 ± 18	36 ± 1	43 ± 11	52 ± 25	n.s.
P_peak_ [cm H_2_O]	17 ± 2	23 ± 5	26 ± 6	16 ± 2	20 ± 4	25 ± 9	n.s.
P_mean_ [cm H_2_O]	9 ± 1	13 ± 3	15 ± 5	9 ± 1	10 ± 3	13 ± 6	n.s.
PEEP [cmH_2_O]	7 ± 1	10 ± 4	12 ± 5	7 ± 1	5 ± 2	9 ± 5	n.s.
ΔP_es_ [cm H_2_O]	0 ± 0	0 ± 2	0 ± 1	0 ± 0	0 ± 1	0 ± 1	n.s.
P_tp_ [cm H_2_O]	7 ± 2	6 ± 1	6 ± 1	6 ± 1	7 ± 1	7 ± 1	n.s.
V_t_ [ml kg^-1^]	6.8 ± 2.1	5.3 ± 2.0	5.5 ± 2.1	6.2 ± 0.5	6.5 ± 0.6	7.1 ± 1.2	n.s.
RR [min^-1^]	36 ± 8	45 ± 5	46 ± 5	39 ± 5	39 ± 15	37 ± 10	p = 0.04
C_dyn_ [ml cm H_2_O^-1^]	32 ± 11	14 ± 8	14 ± 6	33 ± 14	14 ± 4	13 ± 4	n.s.
pH	7.38 ± 0.04	7.17 ± 0.09	7.25 ± 0.08	7.34 ± 0.06	7.21 ± 0.04	7.19 ± 0.11	n.s.
AaDO_2_ [mmHg]	42 ± 19	174 ± 86	229 ± 138	48 ± 26	167 ± 90	214 ± 158	n.s.
etCO_2_ [kPa]	5.5 ± 0.4	5.5 ± 0.5	4.7 ± 0.6	5.3 ± 0.3	6.2 ± 1.1	7.8 ± 4.5	p = 0.02
HR [min^-1^]	106 ± 20	141 ± 27	127 ± 25	103 ± 24	132 ± 22	127 ± 25	n.s.
MAP [mmHg]	96 ± 6	68 ± 11	73 ± 13	90 ± 10	66 ± 7	72 ± 14	n.s.
MPAP [mmHg]	26 ± 6	46 ± 5	38 ± 5	26 ± 4	43 ± 7	35 ± 10	n.s.
CVP [mmHg]	14 ± 3	14 ± 3	15 ± 1	14 ± 1	13 ± 1	14 ± 2	n.s.
CO [L min^-1^]	4.2 ± 0.7	3.1 ± 0.6	2.6 ± 0.6	4.2 ± 1.3	3.3 ± 0.8	3.2 ± 0.7	n.s.
SVR [dyn s cm^-5^]	1570 ± 313	1493 ± 484	1916 ± 417	1547 ± 405	1402 ± 397	1427 ± 185	n.s.
NA [μg kg^-1^ min^-1^]	0 ± 0	0.2 ± 0.3	0.3 ± 0.3	0 ± 0	0.1 ± 0.2	0.2 ± 0.3	n.s.

Data are reported as mean ± SD. Group effects over time are analyzed by two-way-ANOVA and with Holm-Sidak procedure. No intergroup differences during baseline. n.s. = non-significant.

FiO_2_: fraction of inspired oxygen; P_peak_: peak inspiratory pressure; P_mean_: mean airway pressure; PEEP: positive end-expiratory pressure, ΔP_es_: difference of the esophageal pressure; P_tp_: transpulmonary pressure; V_t_: tidal volume; RR: respiratory rate; C_dyn_: dynamic lung compliance; AaDO_2_: alveolar-arterial oxygen difference; etCO_2_: endtidal carbon dioxide; HR: heart rate; MAP: mean arterial pressure; MPAP: mean pulmonary arterial pressure; CVP: central venous pressure; CO: cardiac output; SVR: systemic vascular resistance; NA: noradrenaline dosage.

### Gas exchange and respiratory variables

**Figure 2:**
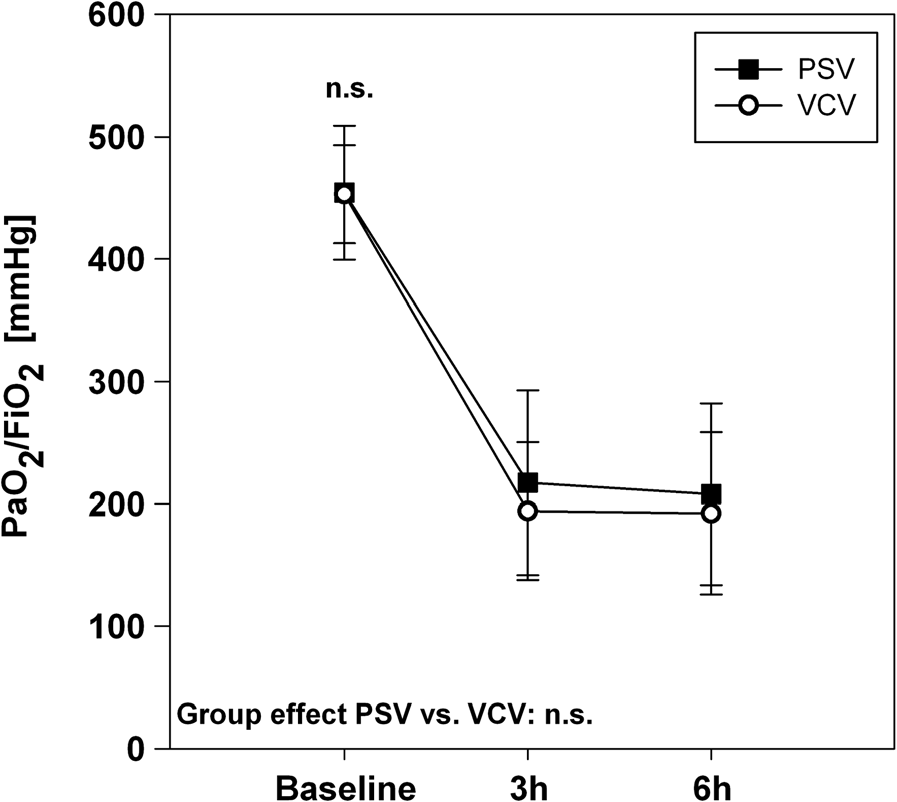
**Quotient of PaO**_**2**_ **and FiO**_**2**_ **(PaO**_**2**_ **/FiO**_**2**_ **).** Group effects over time are analyzed by two-way-ANOVA and post-hoc Holm-Sidak procedure. n.s. = non-significant, PSV: pressure support ventilation, VCV: volume-controlled ventilation.

After sepsis induction both groups developed a ratio of the arterial partial pressure of oxygen (PaO_2_) and FiO_2_ lower than 300 mmHg (Figure 2). Despite higher PaO_2_/FiO_2_ during PSV the values did not reach significance. Tolerable hypercapnia occurred in both groups, which was more pronounced during PSV (p = 0.02) and caused by lower breathing frequencies (p = 0.04). Ventilatory pressures and FiO_2_ had to be raised over time and respiratory mechanics worsened accordingly without group-related differences.

### Regional ventilation distribution and V_A_/Q ratios

**Figure 3:**
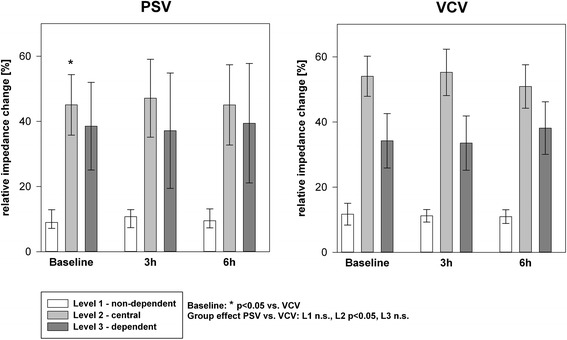
**Regional distribution of the tidal volume [% of the global tidal amplitude] measured by electrical impedance tomography.** *indicates p < 0.05 during Baseline. Group effects over time are analyzed by two-way-ANOVA and post-hoc Holm-Sidak procedure. PSV: pressure support ventilation, VCV: volume-controlled ventilation, L1-L3: Level 1-3.

**Figure 4:**
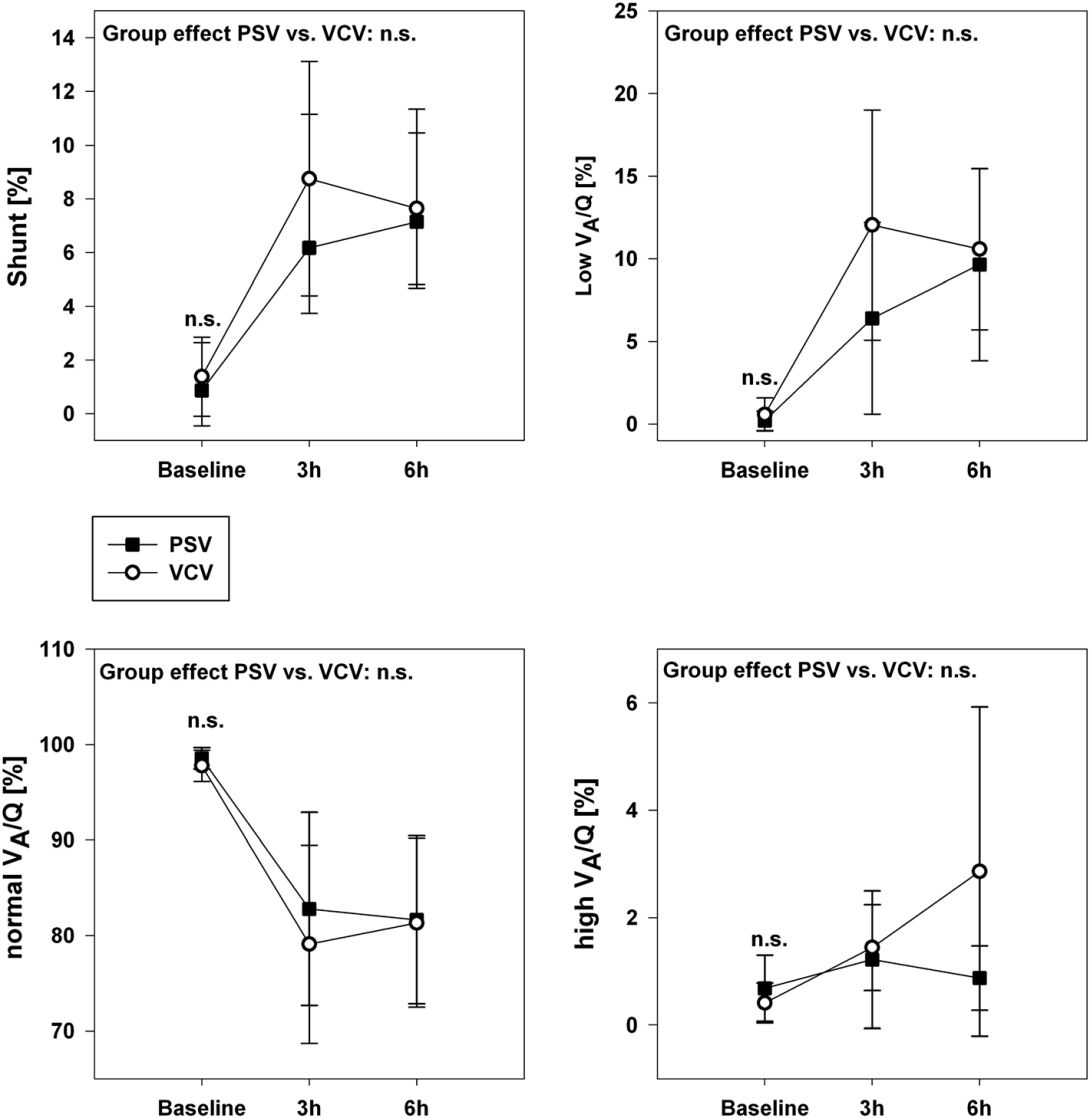
**Ventilation/Perfusion Distribution (V**_**A**_ **/Q) measured by MMIMS-MIGET.** V_A_/Q ratios were defined as follows: shunt (V_A_/Q < 0.005), low V_A_/Q (0.005 < V_A_/Q < 0.1), normal V_A_/Q (0.1 < V_A_/Q < 10), high V_A_/Q (10 < V_A_/Q > 100). Group effects over time are analyzed by two-way-ANOVA and post-hoc Holm-Sidak procedure. n.s. = non-significant, PSV: pressure support ventilation, VCV: volume-controlled ventilation.

Regional ventilation distribution and V_A_/Q were assessed by EIT respectively MMIMS-MIGET at baseline, three and six hours. Starting during baseline the EIT data (Figure 3) show a more homogenous distribution of tidal ventilation between the central and non-dependent lung compartments in the PSV group. The regional ventilation in the central compartment is significantly higher in the VCV-group and correspondingly lower in the dependent area. The V_A_/Q analysis approves healthy baseline conditions. During sepsis increasing shunt and hypoventilated lung areas (low V_A_/Q) represent the main mode of gas exchange impairment (Figure 4). In the early course (hours 1-3) the VCV group tends to develop higher amounts of shunt and low V_A_/Q ratios. After six hours measureable, but still non-significant high V_A_/Q ratios indicating hyperinflation develop in the VCV group.

### Hemodynamics and systemic inflammation

**Figure 5:**
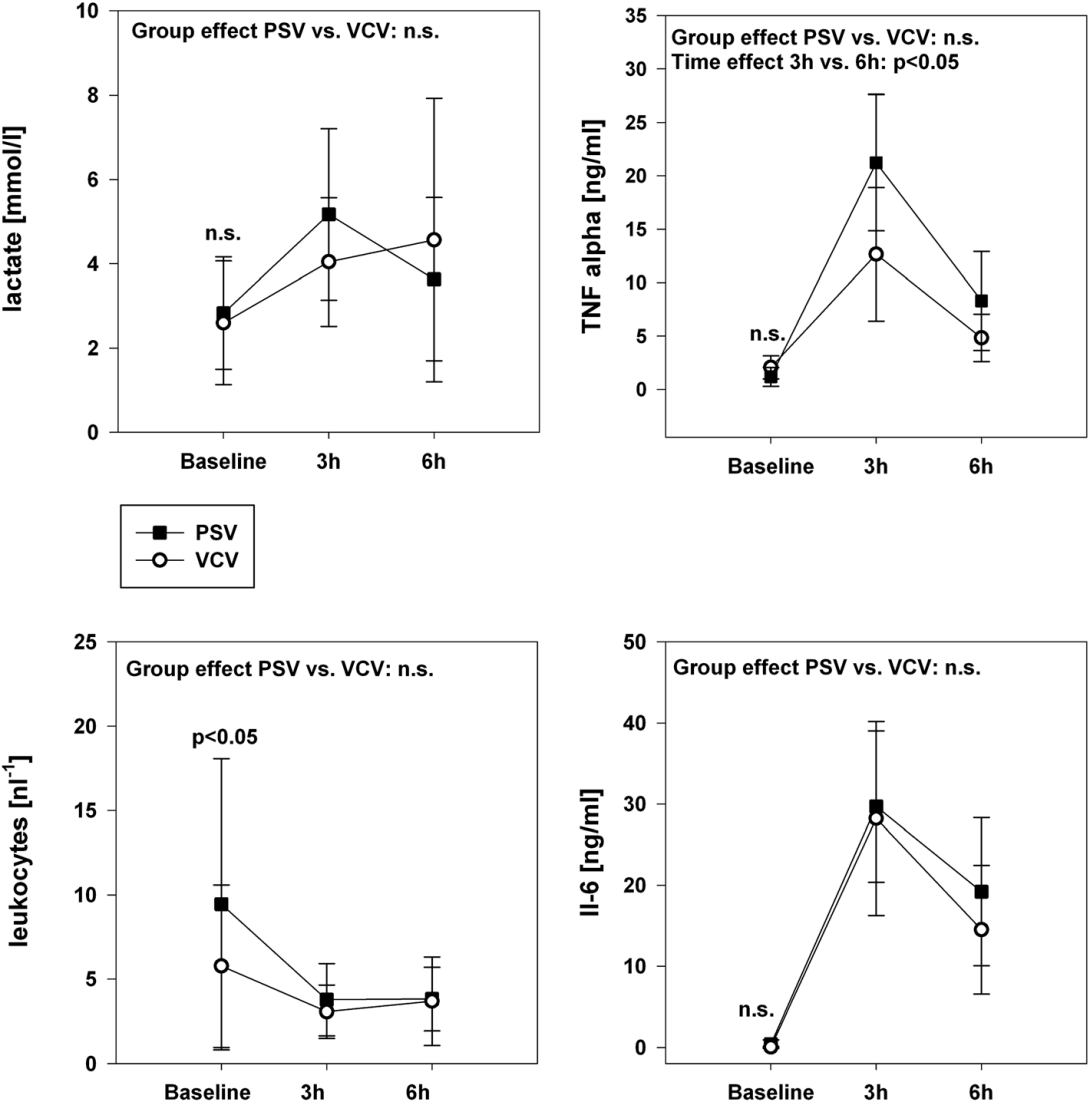
**Hematologic parameters associated with the systemic inflammatory response to LPS administration.** Group effects over time are analyzed by two-way-ANOVA and post-hoc Holm-Sidak procedure. n.s. = non-significant, PSV: pressure support ventilation, VCV: volume-controlled ventilation.

Hypotension, decreased cardiac output, and increased pulmonary arterial pressure required noradrenaline infusion to maintain stable conditions without differences between PSV and VCV. Additionally, both groups received hydroxylethyl starch administration of 389 ± 261 ml (PSV) and 458 ± 428 ml (VCV; p = 0.86). Due to high variances the two groups differ in leucocyte levels during baseline. In both groups the systemic LPS exposition resulted in increasing lactate levels and leucopenia. Peak cytokine levels developed within three hours following the initial high-dose LPS administration and persisted at increased levels over six hours (Figure 5).

### Post mortem analysis

**Figure 6:**
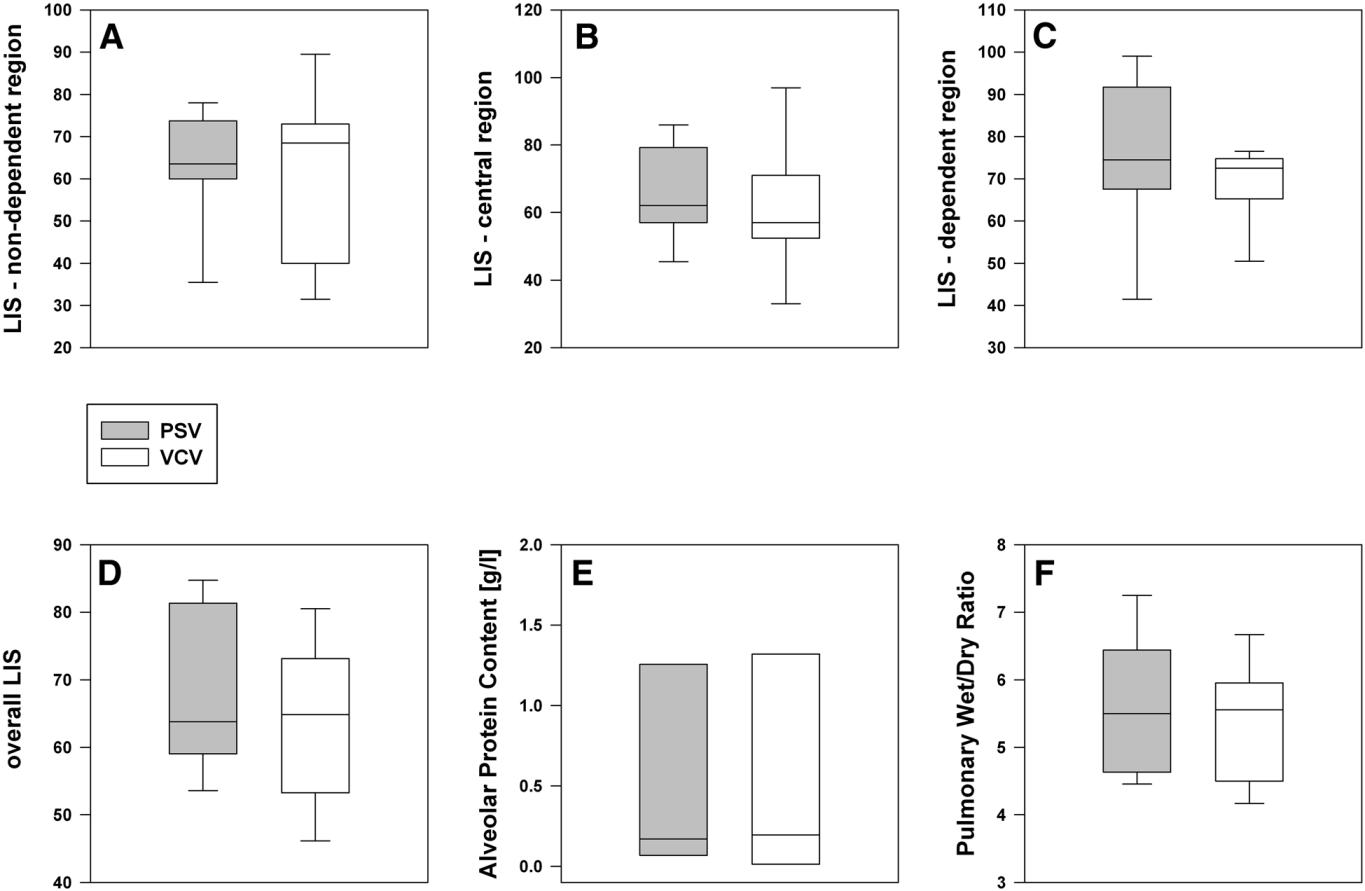
**Post-mortem assessment: histopathological lung injury score (LIS) in different lung regions (A-C) and overall score that represents the mean value of the three region (D), protein content in the bronchoalveolar lavage fluid (E) and pulmonary wet/dry ratio (F).** No significant intergroup differences.

The histopathological analysis approves the presence of sustained lung injury in both groups without intergroup differences or regional variances (Figure 6). In the pooled data from both groups, however, the extent of lung injury is greater in the dependent lung areas (p = 0.048 vs. non-dependent region). The alveolar protein content and pulmonary wet/dry ratio did not differ between the two groups (Figure 6; p = 0.98 respectively p = 0.67).

## Discussion

The present study features the following main findings: in a porcine model of exclusively sepsis-related lung injury the immediate initiation of low V_t_-PSV was feasible, but not superior to low V_t_-VCV in terms of gas exchange, respiratory pattern, hemodynamic stability, and did not improve histopathological parameters over six hours.

Sepsis is one of the most frequent risk constellations of ARDS. Systemic LPS exposition triggers inflammation by releasing pro-inflammatory cytokines and leucocyte accumulation. The early response in experimental models is characterized by acute leucopenia and immense cytokine levels. Hemodynamic findings include decreased systemic blood pressure and pulmonary arterial hypertension [17,18]. Our model adequately reproduces these common early findings. Following central venous LPS infusion the lungs are the first microcirculatory bed to pass. Nevertheless, short-term LPS exposition hardly leads to immediate or persisting ARDS [19,20]. On the other hand, occurrence of severe hemodynamic failure or septic shock conditions limits the systemic LPS application in experimental models. We therefore chose a two-staged infusion regime that caused significant gas exchange deterioration. The reduced maintenance dosage of LPS may also account for a clinically relevant reduction of bacteremia due to therapy. In contrast, primary pulmonary models like bronchoalveolar lavage or acid aspiration rapidly generate atelectasis, reduced lung compliance and gas exchange impairment directly from the beginning on.

The admittance of assisted spontaneous breathing activity can improve the pulmonary function in comparison to conventional lung protective ventilation [6]. Beneficial effects of PSV from several experimental studies amongst others include improved pulmonary blood flow redistribution and overall gas exchange, as well as attenuation of lung injury and IL-6 levels [8,21]. Sophisticated variable pressure support ventilation has shown the potential to further increase these effects in several experimental studies [7,8,22] and is currently tested for clinical application [23,24]. The early use of PSV can decrease sedation requirements, improve the cardiopulmonary function and V_A_/Q matching. The number of days under mechanical ventilation on can also be reduced [25–28]. However, more severely lung injured patients tend to respond poorly to PSV [29]. Additionally, it is reported that early PSV increases patient–ventilator asynchrony [6,30]. This effect may lead to high and harmful tidal volumes even in the early phase of ventilation [31].

Our present findings do not reproduce a significant improvement of gas exchange or lung injury. Ventilation was more homogenously distributed between the central and non-dependent compartment during PSV, but merely a non-significant amount of high V_A_/Q compartments indicating hyperinflation developed in the VCV group. This is a considerable contrast to the upper mentioned results. However, it is worth to take model dependent characteristics into account: for most experimental studies focusing on various assisted spontaneous breathing modes the bronchoalveolar lavage/surfactant-depletion model was used. Furthermore, the assumed mechanisms that mitigate lung injury through spontaneous ventilation vary between several studies and are not fully elucidated [6]. The lavage model immediately induces atelectasis due to surfactant depletion, which are relative easy to recruit in the early phase [29], whereas LPS-injured lungs tend to respond poorly to recruitment strategies [32]. In a rabbit model of lavage-induced lung injury PSV was beneficial only in mild ARDS, though aggravated a pre-existing severe lung injury [33]. The present data, in this context, assume that not only the severity but also etiology and pathophysiologic considerations may considerably influence the response to early PSV. Furthermore, PSV was started before outright fulfillment of ARDS criteria in our model.

Endotoxemia alone causes a relatively moderate V_A_/Q impairment in the short run [34]. This is reflected in the moderate V_A_/Q changes in our data. Interestingly, previous data from a porcine sepsis model combined with non-protective ventilation reported a shunt fraction of 17.3 ± 7.5 [34] without occurrence of low V_A_/Q units, whereas low V_A_/Q units represent the predominant pattern of impairment during our low V_t_ modes (Figure 5). Furthermore, LPS but not bronchoalveolar lavage compromises the hypoxic pulmonary vasoconstriction [35], which should partially compensate the gas exchange deficit. Supporting our results, LPS administration did not significantly affect the ventilation distribution, but only influenced the perfusion pattern [36].

The present study has some limitations: the group sizes were adapted to previous publications that showed beneficial effects of PSV in early ARDS without a prior power analysis. Due absence of a clear-cut trend, however, it appears unlikely that the lack of effect is essentially influenced by the group sizes. We applied standard PSV and not promising but sophisticated spontaneous ventilation approaches like variable, proportional or neutrally adjusted ventilation [6,37]. However, the initiation of early PSV in beginning sepsis should be feasible almost anywhere, not just in specialized intensive care units. Several studies showed that spontaneous ventilation attenuates histopathological lung injury in mild to moderate ARDS models [7,8,33]. But inflammatory response was only slightly altered in comparison to conventional lung protective ventilation [8], while gene expression analysis yielded no significant differences in pulmonary mRNA expression of inflammatory marker genes [7]. With regard to the reported model characteristics and ongoing LPS exposition, which is documented in the high plasma cytokine levels (Figure 3), significant variances in tissue contents of inflammatory markers are highly improbable without the presence of an anti-inflammatory agent over six hours. The present study was designed to focus the early phase of sepsis with a developing lung injury. If possible effects over six hours proceed towards improved long-term outcome, is merely speculative. Nevertheless, adequate identification and selection of patients may considerably influence the effectiveness of early PSV.

## Conclusion

In a porcine model of early LPS-induced lung injury direct initiation of low V_t_-PSV did not improve pulmonary function or affect lung injury in comparison to low V_t_-VCV within six hours. This is a contrast to several studies that report beneficial effects of assisted spontaneous breathing modes in non-septic experimental models of mild to moderate ARDS. Early response to PSV in ARDS seems to be determined not exclusively by severity but also by etiology of the developing lung injury.

## Abbreviations

ANOVA: Analysis of variance
ARDS: Acute respiratory distress syndrome
EIT: Electrical impedance tomography
etCO_2_: Endtidal CO_2_
FiO_2_: Fraction of inspired oxygen
IQR: Interquartile range
LPS: Lipopolysaccharide
MMIMS-MIGET: Micropore membrane inlet mass spectrometry - multiple inert gas elimination technique
PaO_2_: Arterial partial pressure of oxygen
PSV: Pressure support ventilation
PEEP: Positive end-expiratory pressure
V_t_: Tidal volume
V_A_/Q: Ventilation/perfusion distribution
VCV: Volume controlled ventilation
